# Allometry and phylogenetic divergence: Correspondence or incongruence?

**DOI:** 10.1002/ar.25544

**Published:** 2024-07-24

**Authors:** Andrea Cardini

**Affiliations:** ^1^ Dipartimento di Scienze Chimiche e Geologiche Università di Modena e Reggio Emilia Modena Italy; ^2^ Centre for Forensic Anatomy and Biological Sciences The University of Western Australia Crawley Western Australia Australia

**Keywords:** Cercopithecidae, cranium, geometric morphometrics, phylogenetic signal, sampling error, vector angles

## Abstract

The potential connection between trends of within species variation, such as those of allometric change in morphology, and phylogenetic divergence has been a central topic in evolutionary biology for more than a century, including in the context of human evolution. In this study, I focus on size‐related shape change in craniofacial proportions using a sample of more than 3200 adult Old World monkeys belonging to 78 species, of which 2942 specimens of 51 species are selected for the analysis. Using geometric morphometrics, I assess whether the divergence in the direction of static allometries increases in relation to phyletic differences. Because both small samples and taxonomic sampling may bias the results, I explore the sensitivity of the main analyses to the inclusion of more or less taxa depending on the choice of a threshold for the minimum sample size of a species. To better understand the impact of sampling error, I also use randomized subsampling experiments in the largest species samples. The study shows that static allometries vary broadly in directions without any evident phylogenetic signal. This variation is much larger than previously found in ontogenetic trajectories of Old World monkeys, but the conclusion of no congruence with phylogenetic divergence is the same. Yet, the effect of sampling error clearly contributes to inaccuracies and tends to magnify the differences in allometric change. Thus, morphometric research at the boundary between micro‐ and macro‐evolution in primates, and more generally in mammals, critically needs very large and representative samples. Besides sampling error, I suggest other non‐mutually exclusive explanations for the lack of correspondence between allometric and phylogenetic divergence in Old World monkeys, and also discuss why directions might be more variable in static compared to ontogenetic trajectories. Even if allometric variation may be a poor source of information in relation to phylogeny, the evolution of allometry is a fascinating subject and the study of size‐related shape changes remains a fundamental piece of the puzzle to understand morphological variation within and between species in primates and other animals.

## 1 INTRODUCTION

The interaction between ontogenetic and evolutionary change has been a main research area in evolutionary developmental biology for more than a century (Minelli, 2020). Haeckel strongly argued that ontogeny recapitulates phylogeny, thus creating a connection between ontogenetic and evolutionary change (Dayrat, 2003; Kluge & Strauss, 1985), and Gould (1966) stressed the importance of ontogenetic and especially allometric variation in animal evolution. Even if the theory of recapitulation, which predates Haeckel's emphasis on its putative centrality in evolution (Kluge & Strauss, 1985), has been largely disproved (save specific exceptions—Richardson & Keuck, 2002), discussions by Haeckel and Gould made clear the relevance of regional changes in the timing of events during development, when descendants and ancestors are compared (i.e., heterochrony—Hall & Hanken, 2023).

In mammals, and especially in primates (Simons et al., 2020, and references therein) in relation to human evolution (e.g., Bastir et al., 2007; León & Zollikofer, 2001; Mitteroecker et al., 2004; Penin et al., 2002), there has been a great interest in understanding whether important aspects of morphological evolutionary variation might originate, at least to a certain degree, from heterochronic changes. For instance, an extension or truncation of a common ontogenetic trajectory, via selection on adult size, may have produced differences thanks to a small change in the regulatory genes controlling the endpoint of ontogeny. More realistic heterochronic changes, however, are likely to happen by simultaneous modification of multiple parameters (not only extension or truncation, but also rates of development, etc.) in ontogeny (Kluge & Strauss, 1985). Heterochrony can be investigated experimentally in a number of model organisms (Dobreva et al., 2022), but it can also be explored using morphometrics, the quantitative study of morphological differences (Rohlf, 1990). Within the popular framework of Procrustean geometric morphometrics (Adams et al., 2004), there are a variety of approaches available to dissect and compare the specific aspects of morphological trajectories and heterochronic change (Klingenberg, 2016; Mitteroecker et al., 2013; Piras et al., 2016; Sheets & Zelditch, 2013; Simons et al., 2018).

The proportions of an organism (i.e., its shape) often vary in relation to size, a type of covariation called allometry (Klingenberg, 1998, 2022). Allometry may occur at different levels, but it is most evident in ontogeny and evolution. Human babies, for instance, are born with a relatively large head, since the brain and sensory organs develop early. As the rest of the body grows faster after birth, however, adults end up with a proportionally smaller braincase and both a longer face and body (Lieberman, 2011). This type of ontogenetic allometry is probably, in broad terms, a rule with few exceptions among placentals (Smith, 1997). Likewise, although the mechanisms are unclear, within a clade of closely related species of terrestrial vertebrates, evolutionary divergence frequently occurs so that larger species tend to have proportionally longer snouts but shorter braincases, a macroevolutionary pattern known as “CRanial Evolutionary Allometry” (CREA—Cardini, 2019; Cardini & Polly, 2013; Krone et al., 2019; Marugán‐Lobón et al., 2022; Radinsky, 1985). Beside ontogenetic and evolutionary, there is a third kind of allometric variation, called “static.” With static allometry, when present, intraspecific size‐related shape variation happen within a given ontogenetic stage (e.g., adults of the same species).

Allometric patterns can be more or less conserved in evolution, and allometry can be both a constraint and an accelerator of morphological change (Gould, 2002; Kluge & Strauss, 1985; Pélabon et al., 2014). Ontogenetic and evolutionary allometry are of particular interest in evolutionary developmental biology (Klingenberg, 2010), the discipline that studies the relationships between evolution and development: “From the point of view of evolutionary developmental biology (evo‐devo), evolvability is largely a function of developmental systems' ability to generate variation (Hendrikse et al., 2007). Through development, genetic variation is translated into phenotypes subject to what has been called developmental constraint or bias [sic], which include modularity, canalization, heterochrony, allometry, and integration” (p. 218, Minelli, 2009). Data on static allometry, however, also have a role to play “because it is the level at which developmental constraints can be easily measured” (p. 62, Pélabon et al., 2014), thus contributing to generate predictions about phenotypic evolution. In practice, static allometries not only are informative in relation to evolutionary change, but, when ontogenetic series are not available, can also provide clues on ontogenetic allometries. This is because, even if there can be differences, static patterns of size‐related variation are a consequence of growth and development during ontogeny (Pélabon et al., 2014).

In this article, I explore the potential connection between static allometry, an aspect of microevolutionary variation, and interspecific divergence within an evolutionary radiation. The primary hypothesis is whether there is any degree of congruence between the change in direction of interspecific allometries and phylogeny. A number of previous studies (e.g., Pavón‐Vázquez et al., 2022; Simons et al., 2018, 2020) have suggested that variation in allometric direction (measured by regression slopes) might be the most variable aspect in the evolution of allometric patterns. Divergence in allometries, however, can happen (if it does) in different modalities. If the relationship between shape and size variation is progressively modified as species evolve, a prediction that angles in allometric trajectories should increase proportionally to phylogenetic distances may be tested. Therefore, for instance, in relation to the taxonomic hierarchy in phylogenetic systematics, differences in allometric directions should be smaller between species of the same genus, larger between species of different genera and even larger across tribes and families. Alternative hypotheses are that allometries could be highly conserved and hardly change or, at the opposite extreme of variability, be so labile that the rate of divergence varies widely; in both instances, the phylogenetic signal is likely to be tiny or totally lost.

The study group for my analysis is the Old World monkeys (family Cercopithecidae—Groves, 2001), whose African origin dates between 25 and 35 million years ago, although most of the living species belong to more recent, and still ongoing, radiations occurring in the last 10 million of years (Frost, 2017). Modern cercopithecids are diverse. They show wide interspecific differences in body mass, ranging from a small talapoin of about 1 kg to a large male mandrill or baboon weighing 30 kg or more. Likewise, they have large variability in diet, habitat and, more generally, ecology (Elton, 2007). This lineage also offers textbook examples of surprising disagreements between morphological and DNA analyses, such as mangabeys or baboons‐mandrills not being monophyletic despite very strong phenotypic similarities (Collard & O'Higgins, 2001; Gilbert & Rossie, 2007; Harris, 2000, 2002; Lycett & Collard, 2005; Singleton, 2002). To better understand the mechanisms that seem to have almost decoupled morphological and molecular evolution in some of the Old World monkeys, the Cercopithecidae have been the subject of numerous evo‐devo studies using morphometrics (Simons et al., 2018; Simons & Frost, 2021; Singleton, 2012, and references therein).

**FIGURE 1:**
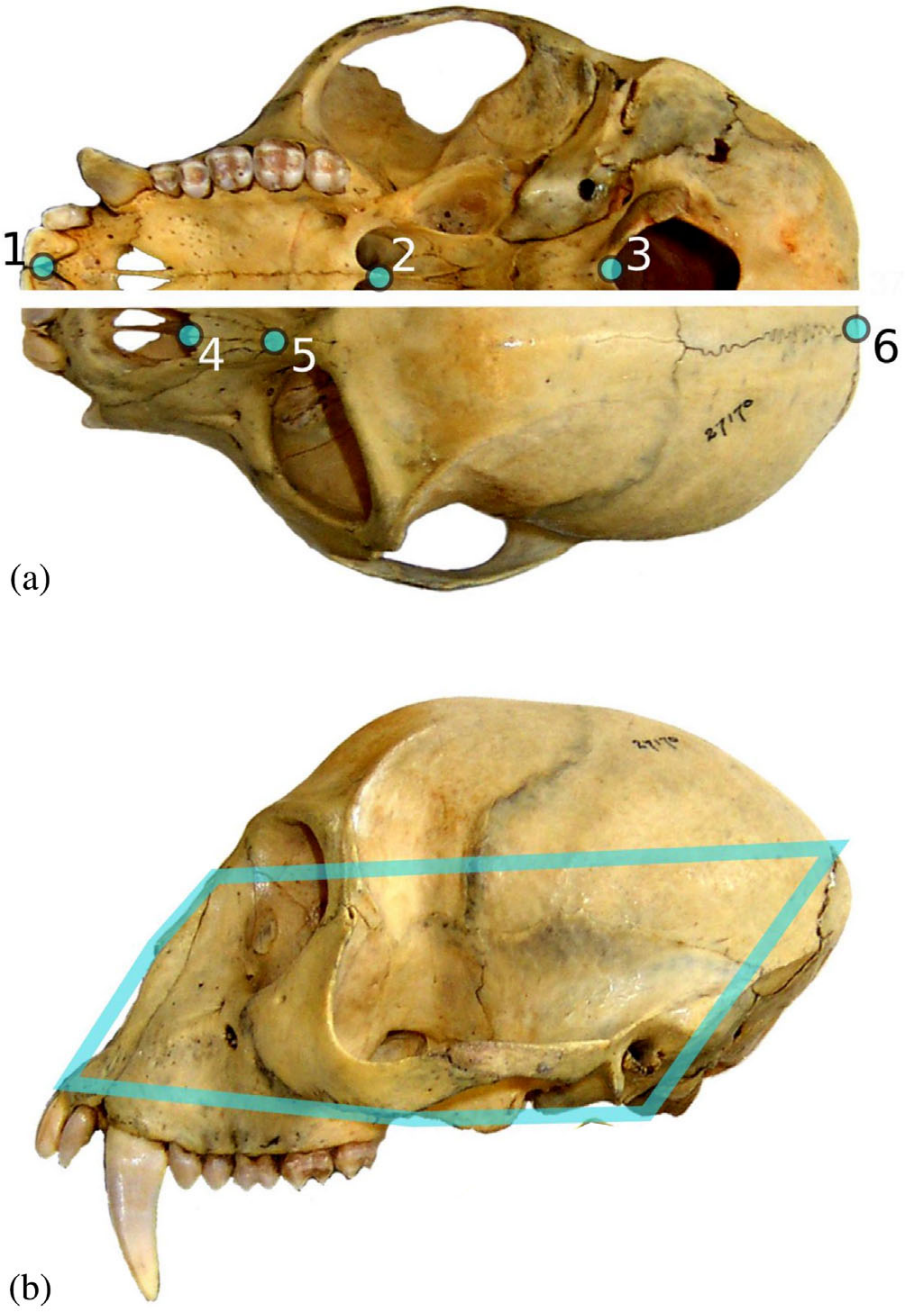
Landmark configuration (a) and wireframe (b). The definitions of the landmarks are: 1, prosthion, antero‐inferior point on the projection of pre‐maxilla between central incisors; 2, palatine posterior edge on the midline; 3, basion, anterior‐most point of the foramen magnum; 4, rhinion, most anterior midline point on nasals; 5, nasion, midline point on fronto‐nasal suture; 6, inion, most posterior point of the cranium in dorsal view.

It is in this specific context that I have explored whether the divergence in static allometry and the phylogenetic divergence show correspondence or incongruence. The original dataset consisted of more than 3200 adult crania of 78 species of cercopithecids (Table 1). However, to mitigate against inaccurate estimates in small samples, I excluded the species with fewer individuals and selected for the study only 51 species with larger samples, for a total of almost 3000 specimens. Cranial size and shape were, thus, analyzed using Procrustean geometric morphometrics and a small set of well‐defined midplane cranial landmarks (Figure 1). This landmark configuration is simple, but it measures an important aspect of craniofacial allometric change in the ontogeny and evolution of mammals and other terrestrial vertebrates (Cardini, 2019; Radinsky, 1985; Smith, 1997), which is the relative length and depth of the snout and braincase in relation to cranial size.

**TABLE 1:** Sample composition. Species samples with less than 10 individuals per sex were not used for the analyses. In this and other tables, F is the abbreviation for females and M for males.

Lineage	Genus	Species	F	M
African Colobini	*Colobus*	*angolensis*	4	4
		*guereza*	21	18
		*polykomos*	9	10
	*Piliocolobus*	*badius*	49	30
		*bouvieri*	1	1
		*ellioti*	65	44
		*epieni*	1	1
		*foai*	12	34
		*gordonorum*	4	0
		*kirkii*	32	10
		*oustaleti*	35	40
		*parmentieri*	45	21
		*preussi*	31	8
		*rufomitratus*	5	1
		*temminckii*	11	5
		*tephrosceles*	8	17
		*tholloni*	38	16
		*waldroni*	15	6
	*Procolobus*	*verus*	20	6
Cercopithecini	*Allochrocebus*	*lhoesti*	16	17
		*preussi*	3	5
	*Allenopithecus*	*nigroviridis*	6	8
	*Chlorocebus*	*aethiops*	11	6
		*cynosuros*	19	15
		*djamdjamensis*	8	6
		*pygerythrus*	51	74
		*sabaeus*	11	30
		*tantalus*	17	23
	*Cercopithecus*	*ascanius*	33	37
		*campbelli*	32	29
		*cephus*	27	28
		*diana*	29	32
		*erythrogaster*	4	5
		*erythrotis*	4	10
		*hamlyni*	13	15
		*mitis*	67	78
		*mona*	15	19
		*neglectus*	23	27
		*nictitans*	23	23
		*petaurista*	15	25
		*pogonias*	37	38
		*sclateri*	5	6
	*Erythrocebus*	*patas*	9	16
	*Miopithecus*	*ogouensis*	16	11
		*talapoin*	2	3
Papionini	*Cercocebus*	*atys*	30	23
		*galeritus*	19	27
		*torquatus*	9	14
	*Lophocebus*	*albigena*	23	19
		*aterrimus*	19	21
	*Macaca*	*arctoides*	10	11
		*assamensis*	12	19
		*cyclopis*	14	12
		*fascicularis*	184	281
		*fuscata*	14	11
		*hecki*	9	9
		*leonina*	10	8
		*maura*	3	5
		*mulatta*	36	23
		*nemestrina*	15	18
		*nigra*	13	13
		*ochreata*	3	2
		*pagensis*	2	1
		*radiata*	9	8
		*silenus*	4	3
		*sinica*	6	14
		*sylvanus*	11	3
		*thibetana*	3	7
		*tonkeana*	8	5
	*Mandrillus*	*leucophaeus*	21	15
		*sphinx*	6	9
	*Papio*	*anubis*	54	123
		*cynocephalus*	11	59
		*kindae*	11	11
		*hamadryas*	4	19
		*papio*	1	12
		*ursinus*	8	44
	*Theropithecus*	*gelada*	14	16

In the study, I have also investigated the impact of sampling error on the estimates of the direction of static allometries, a central issue for answering the main study question on allometric and phylogenetic divergence. This type of sensitivity analysis was performed using randomized subsampling experiments in the species with the largest sample sizes. Interspecific analyses in morphometrics are often done using relatively small numbers of specimens with little attention to the potentially severe inaccuracies introduced by small and sometimes biased samples. Yet, there is an increasing amount of evidence (Cardini et al., 2021, and references therein) that not only intraspecific comparisons of small differences, but also investigations at boundary between micro‐ and macro‐evolution require large and representative datasets for obtaining robust and really meaningful results. Without such accurate findings, we cannot trust the answers we obtain as we try to elucidate the connection between the origin of differences at population level and those generated by speciation processes in primates and other mammals.

## 2 MATERIALS AND METHODS

### 2.1 Dataset

The total dataset included 3221 adult specimens of 78 species of Old World monkeys (Table 1), of which 2942 specimens of the 51 species with at least 10 individuals in one or the other sex were selected for the analysis. The vast majority (97%) are wild individuals collected during the 21st century. Virtually all specimens are from museums (see the supplementary online material for more details) and precisely from the American Museum of Natural History (NY), Academy of Natural Sciences of Philadelphia, UK Natural History Museum (London), Field Museum of Natural History (Chicago), Museum of Comparative Zoology (Harvard), Museum für Naturkunde (Berlin), Zoologische Staatssammlung München (Munich), Kosmos Museo di Storia Naturale dell'Università di Pavia (Pavia), University of Cambridge Zoology Museum (Cambridge, UK), Powell‐Cotton Museum (UK), Hunterian Museum of the Royal College of Surgeons (London), Royal Museum for Central Africa (Tervuren), Staatliches Museum für Naturkunde (Karlsruhe), Senckenberg, Naturmuseum Frankfurt, US Natural History Museum (Washington DC), Anthropology Collection (University of Zurich). The data were borrowed from those used in a series of studies on patterns of morphological variation and evolutionary allometry in placental mammals (Cardini, 2019; Cardini et al., 2021). Those papers provide more information on samples, as well as on measurement error in a larger configuration (Cardini & Elton, 2008a) that includes the landmarks employed in this study.

The classification largely followed Groves' (2005) assessment of primate taxonomy in the *Mammal Species of the World* (Wilson & Reeder, 2005). There were a few exceptions, however, such as the distinctively small *Papio kindae*, a subspecies of *P. cynocephalus* in Groves (2005), but a valid species in this study as well as in recent revisions (Burgin et al., 2018; Mammal Diversity Database, 2023). Also, with some of the red colobus (genus *Piliocolobus*), whose taxonomy remains unstable and subject to frequent changes (Oates & Ting, 2015), I generally followed the classification used in the original museum database. This mostly means that some of the populations classified as subspecies by Groves (2005) are raised to species level, that is similar to the taxonomic revision of *Piliocolobus* in the Mammal Diversity Database. However, in a couple of cases, the museum classification at the time of data collection recognized as a valid species a taxon, which is now seen as dubious. To avoid potentially incorrect updates, I followed the museum original taxonomy and, therefore, analyzed as separate taxa both *P. ellioti*, now seen either as a synonym of *P. semlikiensis* or a hybrid between other red colobus species (Maisels & Ting, 2020), and *P. temminckii*, possibly a synonym of *P. badius* (Mammal Diversity Database, 2023) or one of its subspecies (Groves, 2005). Therefore, because of taxonomic inaccuracies, as well as the smaller number of species, which only represent part of the African radiation of colobine monkeys, results for the Colobini are largely preliminary and must be interpreted with caution.

### 2.2 Analysis samples and landmark configuration

I performed all analyses using separate sexes, because of the variable but usually large sexual dimorphism of Old World monkeys (Cardini & Elton, 2008c; Plavcan, 2001). This decision has the disadvantage of reducing sample size (N), but allows to have more homogeneous samples, where variation due to static allometry is not mixed up with sex differences in adults. Estimates of static allometries, however, can be severely affected by sampling error (Cardini & Elton, 2007). Thus, species with very small samples must be excluded. An inevitably arbitrary decision of a minimum N for inclusion is not straightforward and has an impact on taxonomic sampling: with a smaller minimum N, more species are included but species with small samples have large inaccuracies; with larger N, there are fewer species in the analysis but estimates of their allometric slopes should be more accurate. To explore the consequences of this trade‐off, within each sex I selected species with at least 10, 20, or 30 specimens and, after re‐superimposing the data, repeated all analyses using each of the three different thresholds for minimum N.

The landmark configuration is shown in Figure 1. All landmarks are on the midplane and small asymmetries orthogonal to this plane in the original set of 3D landmarks have been removed following Cardini (2017). Thus, the six midplane landmarks are, in fact, two‐dimensional (as if they had been digitized on a photograph) and the specimens lie in an eight dimensional shape space, after accounting for the loss of degrees of freedom in the Procrustes superimposition (see next subsection). The raw coordinates of the whole sample are available as supplementary online material in the same spreadsheet as Tables S1 and S2. A detailed list of the software for the analyses described in the next subsections can be found at the end of the methods.

### 2.3 Geometric morphometrics: Size and shape variables

I computed the centroid size (CS) of each configuration and the matrix of shape coordinates using a Procrustes superimposition (Goodall, 1991; Gower, 1975; Rohlf & Slice, 1990; Sneath, 1967). For testing allometries, I transformed CS using the natural logarithm (lnCS) and used the non‐zero eigenvalue principal components (PCs) of the shape coordinates in the regressions, always including all eight PCs. This is equivalent to using the full matrix of Procrustes shape coordinates, but has the advantage of removing the redundancy introduced by the loss of degrees of freedom after the superimposition. Thus, the full shape information is used in all analyses, but none of the parametric tests is impacted by potentially inaccuracies that can happen when the software miscalculates the degrees of freedom of the data.

Subsamples of shape variables projected in the tangent space, as required by most statistical analyses (Klingenberg, 2020), might slightly distort the Procrustes shape distances. I anticipate here, in the methods, that in the total sample (*N* = 3221), as well as in each of the six subsamples (females and males with *N* ≥10 or 20 or 30), distortions were totally negligible. This was assessed by plotting pairwise Euclidean shape distances computed using the full set of eight PCs of a subsample projected in the tangent space and the corresponding pairwise Procrustes shape distances. Also, I computed the matrix correlation between the two sets of distances matrices. In all plots, the deviation from a straight line with slope one, passing through the origin of the axes, was always minuscule (not shown) with matrix *r* > 0.99.

### 2.4 Allometries

I tested static allometry within each species using a multivariate regression of all eight shape PCs onto lnCS (Klingenberg, 2022), computed the multivariate variance accounted for by the predictor (R squared, abbreviated as Rsq) and estimated the significance of the regression (parametric F approximation for the Wilks' lambda). For the sake of brevity, from now on, I use interchangeably the terms static allometric trajectory and static allometry to refer to the regression line estimated by the multivariate regression. Also, I will call “non‐negligible” any allometric regression that meets the criterion of being either significant (*p* < 0.005, following Benjamin et al., 2018) or of having Rsq >0.05 (5% of shape variance accounted for by allometry). Because Rsq tends to be overestimated in smaller samples (Cramer, 1987), it must be used with caution, but has the advantage of being easy to interpret. The thresholds I am using (*p* and Rsq) are both arbitrary, but help to filter out the cases with the weakest evidence for static allometry. In this respect, 5% of shape variance accounted for by allometry may seem like a small amount, but static allometry in adult primates of the same sex is unlikely to have a very large effect and 5% is about the same as the Procrustes shape variance explained by biologically meaningful variation in crania such as, for instance, sex differences in human adults (Bigoni et al., 2010; Del Bove et al., 2020; Franklin et al., 2012). Setting *p* < 0.005 reduces the risk of false positives including, to a degree, those related to the potential inflation of type I errors in multiple tests.

Using the results of the multivariate regressions, I selected the species with non‐negligible allometries in each sex and dataset (minimum *N* ≥ 10, 20, or 30), and measured the divergence in allometric directions by computing pairwise angles between the vectors of slope regression coefficients. To this aim, I employed the formula for the inner dot product of a pair of vectors and converted the result from radians to degrees. I also visually summarized allometries using a scatterplot of predicted allometric shapes onto lnCS. The predicted shapes were first subjected to a PCA (Adams & Nistri, 2010) and then the resulting PCs rotated to maximize their covariation with lnCS, as it is done to obtain regression scores (Drake & Klingenberg, 2008). This further rotation may improve the accuracy of the visualization of the predictions in relation to the predictor (lnCS, in this case) or leave the scores almost unchanged compared to those of PC1 of the predictions.

### 2.5 Congruence between allometric and evolutionary divergence

**FIGURE 2:**
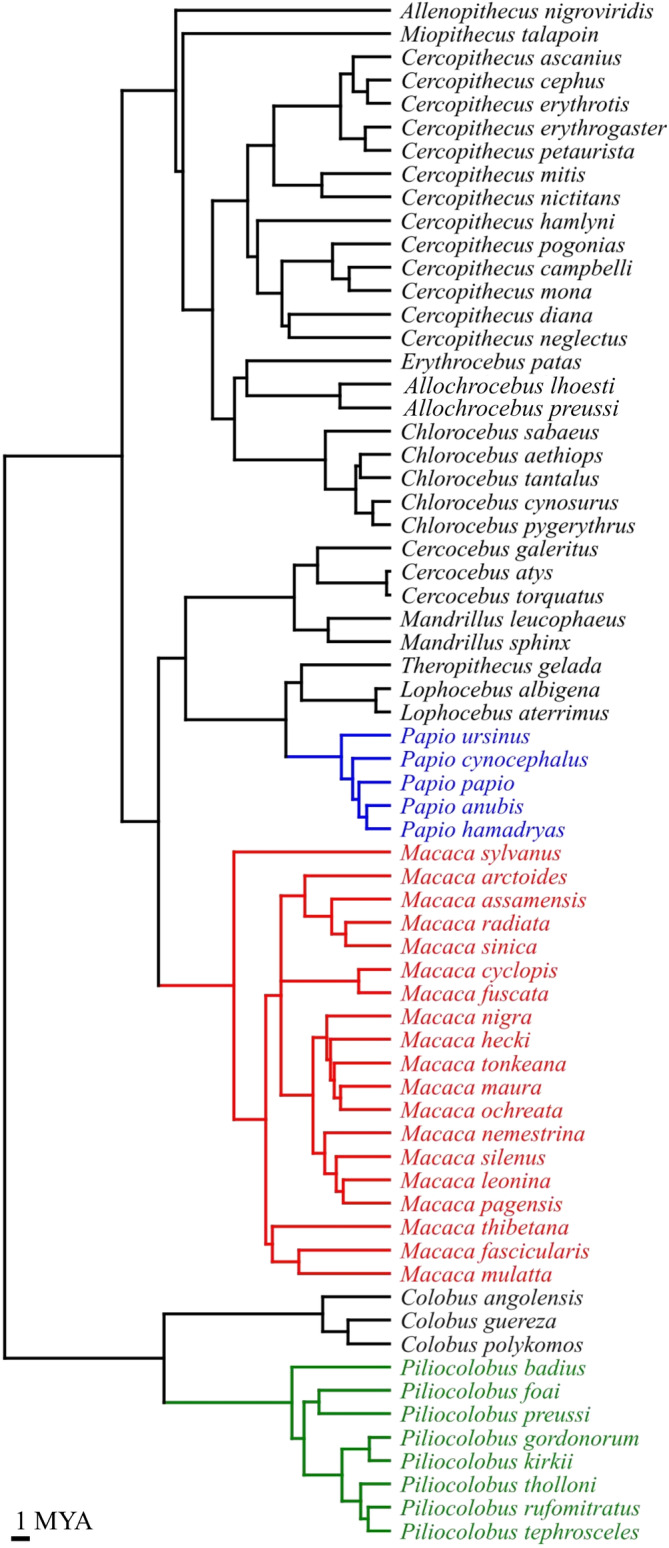
10KTrees consensus chronogram (https://10ktrees.nunn‐lab.org/) for most of the Old World monkey species in the study (MYA, millions of years ago). Three of the most diverse monophyletic genera are emphasized using colors. These three genera are later used (Figure 5) as examples to explore phylogenetic clustering of allometric trajectories.

The main part of the study is the analysis of the pattern of allometric divergence in relation to phyletic relationships. This requires an independent phylogeny (unrelated to cranial variation) to be used as a proxy for the evolutionary history of the lineage. For the phylogenetic framework (Figure 2), I chose the consensus chronogram of the 10Ktrees molecular phylogeny of Arnold et al. (2010). The phylogeny is mainly based on mitochondrial DNA, includes most of the species in my analysis and corroborates the monophyly of the majority of the traditional genera of Old World monkeys (e.g., *Chlorocebus*, *Macaca*, *Papio*, *Piliocolobus*). The main exception is *Cercopithecus*. Among species available in my study, two were formerly recognized as *Cercopithecus* (*C. lhoesti* and *C. preussi*), but now they form a separate clade, the genus *Allochrocebus*, which is closer to *Chlorocebus* and patas monkeys. Samples of these two species, however, were modest or small in my dataset and, therefore, only *A. lhoesti* was included in the analyses using the smallest N threshold (≥10).

To explore the congruence between allometric and evolutionary divergence I used multiple approaches, which partly overlap with those of Watanabe and Slice (2014). The first is purely graphical and uses an Unweighted Pair Group Method with Arithmetic Mean (UPGMA) phenogram to summarize interspecific similarity relationships based on the pairwise angles of allometric trajectories. This is also similar to Simons et al.'s (2018) use of a PCA to summarize the coefficients of cranial ontogenetic trajectories. A very basic expectation for a degree of congruence with phylogeny is that allometric angles will produce clusters of species corresponding to monophyletic genera. Likewise, angles between species of the same genus should on average (median angle) be smaller than angles between species of different genera. If the divergence in allometric trajectories increases with phyletic distance, pairwise interspecific angles should also be correlated to patristic distances calculated using the molecular phylogeny and, thus, have a high matrix correlation. In this specific analysis, however, I did not test the significance of the matrix correlation, which is a crude way to assess congruence between phenotypic and phylogenetic data and has potential statistical issues (see Watanabe & Slice, 2014, and references therein). In contrast, for assessing the significance of the phylogenetic signal, I employed *K*, the multivariate extension of Blomberg k statistics (Adams, 2014; Blomberg et al., 2003) with a 1000 randomization test that permutes the shape data among the tips of the phylogeny. *K* calculates the degree of interspecific similarity in a set of measurements (allometric trajectories, in this study) compared to the similarity expected from a Brownian motion model of trait evolution according to phylogeny (the 10Ktrees chronogram, in my case). With *K* = 1, the measurements match the expectations of the model (i.e., the correspondence between allometric and evolutionary divergence is perfect); if *K* < 1 the correspondence with the phylogeny is poor; if *K* > 1 the direction of allometries is more similar among closely related species than expected from the phylogeny. As the test is only assessing whether *K* is significantly larger than it would be in the absence of any phylogenetic signal, however, one cannot conclude that *K*, even when significant, does not deviate significantly from one. Thus, significance indicates that a degree of correspondence between allometric and phylogenetic divergence is found, which is unlikely to be present by chance given those samples and data. However, this does not say whether *K* is significantly less or more than one. Also, because the computation of *K* cannot be done directly using the matrix of pairwise interspecific angles of allometric trajectories, I first run a principal coordinates analysis on the matrix of pairwise angles and then used the full set of PCOAs to compute K. Finally, Blomberg et al. (2003) warned that the random permutation approach has low error and good power for samples of 20 species or more, which, if applicable also to the multivariate extension of *K*, might contribute to explain some of the similarities in the results of analyses using *N* ≥ 20 and *N* ≥ 30 (see below).

### 2.6 Randomized subsampling experiments to explore the impact of sample size on estimates of angles between allometric trajectories

As in all other analyses, I kept females and males separate, but I limited this part of the analysis to the species with non‐negligible allometric regressions in the largest samples (*N* ≥ 30), which were six for females and nine for males (see Table 2 in the Results). For each of these species, I first bootstrapped the total sample and then randomly selected either 30, 20, or 10 specimens. The design of the analysis is, thus, perfectly balanced (i.e., *N* is identical in each species). In the next paragraph, I use *N* = 30 in the females as an example to describe how bootstrapped subsamples were analyzed to explore the impact of N on angles of allometric trajectories. The same procedure was repeated with *N* = 20 and with *N* = 10. After females, I did the same series of randomized bootstrapped subsampling with *N* = 30 or 20 or 10 in males.

**TABLE 2:** Summary statistics for the species pairwise angles of static allometries. In all tables, angles are in degrees. Abbreviations (valid also for other tables): Species *n* = number of species with non‐negligible static allometries; min *N* = minimum species sample size; SD = standard deviation; q10 and q90 are, respectively, the 10th and 90th percentiles; min and max are, respectively, the minimum and maximum.

Sex	Species *n*	Min. *N*	Mean	SD	Median	q10	q90	Min	Max
F	6	30	56	19	53	31	80	24	88
	9	20	54	19	54	32	79	16	88
	31	10	72	23	70	43	104	16	143
M	9	30	62	25	60	36	91	15	134
	16	20	59	19	58	37	83	15	134
	40	10	70	23	67	41	101	15	143

With the six bootstrapped species samples of females with *N* = 30, I did the multivariate allometric regressions of shape (always all eight PCs) onto lnCS and computed the species pairwise angles between vectors of slope coefficients, as in the main analysis (2.4). In this example, because there are six species, there will be 15 pairwise angles. The computation (regressions and angles) was repeated 100 times, each time redoing the bootstrap and random selection of 30 individuals per species. To obtain an average result, I used the median of the 100 estimates of each of the 15 pairwise angles. Finally, to compare this averaged outcome with the angles estimated for the same species in the main analysis (2.4), I computed the difference between the 15 bootstrapped subsamples median angles and the corresponding 15 observed angles in the original total samples. Thus, negative or positive differences mean that on average angles in samples with *N* = 30 are, respectively, under‐ or over‐estimated relative to those in the main analysis using all individuals. To concisely report the 15 differences, I used a set of summary statistics (mean, standard deviation, range, etc.).

### 2.7 Software

I performed most analyses in R (R Core Team, 2023) using a range of packages and custom scripts with some of the code modified from the one written by chatGPT (OpenAI, 2022) accessed in January/February 2024. The main software/packages in the study were:

1. Morpho (Schlager, 2017) for the Procrustes superimposition and the computation of regression scores.
2. car (Fox & Weisberg, 2019) for multivariate regressions of the eight PCs of Procrustes shape coordinates onto lnCS (some computations were double checked in vegan—Oksanen et al., 2011).
3. ggplot2 (Wickham, 2011) for drawing scatterplots.
4. ape (Paradis et al., 2004) and TreeView 1.6.6 (Page, 1996) for tree editing and drawing.
5. geomorph (Adams & Otárola‐Castillo, 2013) for computing and testing *K*.
6. TPSRegr (Rohlf, 2015) to further double check multivariate regressions and mainly for the visualization of allometric shape changes using wireframes and TPS deformation grids (Klingenberg, 2013).
7. MorphoJ (Klingenberg, 2011) to replicate and check some of the analyses done in R (e.g., PCA, angles between allometric trajectories).

Other R functions, such as those used to compute PCAs and PCOAs, phenograms, cophenetic distances, bootstrapped subsamples, and so forth belong to the base and stats packages of R (R Core Team, 2023).

## 3 RESULTS

### 3.1 Allometries

Because the regression analyses of species samples with a larger minimum *N* should produce, in relative terms, the most accurate estimates, I present first the results for the species with *N* ≥ 30, followed by those with *N* ≥ 20 and *N* ≥ 10. Detailed numerical statistics for the regressions are in the Table S1 and S2 and summarized in words in the next paragraph.

In females, depending on the minimum *N*, there were 6 (*N* ≥ 30), 9 (*N* ≥ 20), and 31 (*N* ≥ 10) non‐negligible static allometric regressions (Table S1). In males, the corresponding numbers of non‐negligible allometric regressions (Table S2) were 9 (*N* ≥ 30), 16 (*N* ≥ 20) and 40 (*N* ≥ 10). In the species with non‐negligible regressions, Rsq ranged overall from 4 to 31%, with a median of 11%, in females, and from 5 to 27%, with a median of 10%, in males. In both sexes, however, Rsq tend to be slightly smaller (7–8% using medians, respectively, in females and males) if only the larger samples (*N* ≥ 30) are considered. The proportion of statistically significant (*p* < 0.005) regressions ranged in females from 83% (*N* ≥ 30) to 19% (*N* ≥ 10) and in males from 67% (*N* ≥ 30) to 27% (*N* ≥ 10). As anticipated in the methods, both positively biased Rsq and a reduction in statistical power, with proportionally fewer significant tests, were expected when smaller samples are included.

**FIGURE 3:**
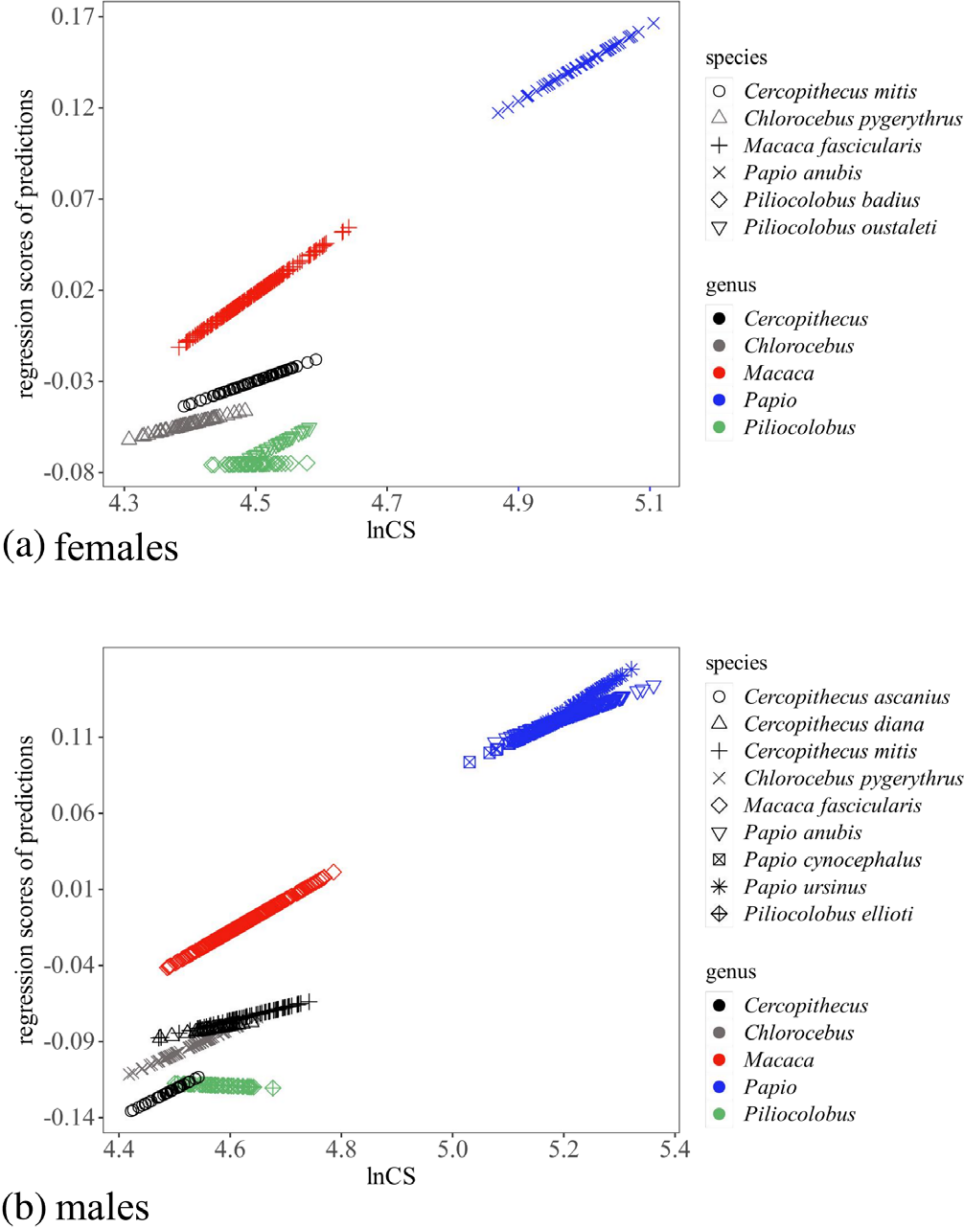
Summary scatterplots of non‐negligible static allometric trajectories in the larger samples (*N* ≥ 30) of females (a) and males (b): lnCS is on the horizontal axis and a summary of the allometric predictions (see Sections 2 and 3) is on the vertical axis.

As an example, non‐negligible allometric trajectories of females and males in species with *N* ≥ 30 are shown in Figure 3. The scatterplots mainly illustrate the wide range of adult size variation and suggest some differences among species, such as the apparently more horizontal scatter of some of the red colobus. However, as I clarify later (see Section 4), sampling and dimensionality reduction may impact these summary plots, which should be interpreted with caution. Thus, on the horizontal axis (lnCS), larger variation in size (as seen, for instance, in *M. fascicularis*) tends to be associated with larger N. On the vertical axis (the summary of allometric predictions), differences in trajectories are potentially interesting, but this axis summarizes 86–95% (respectively, in females and males) of the variance in allometric predictions. This implies that, even when trajectories appear to point to a similar direction, further allometric divergence is likely to be present in the remaining allometric data space.

**FIGURE 4:**
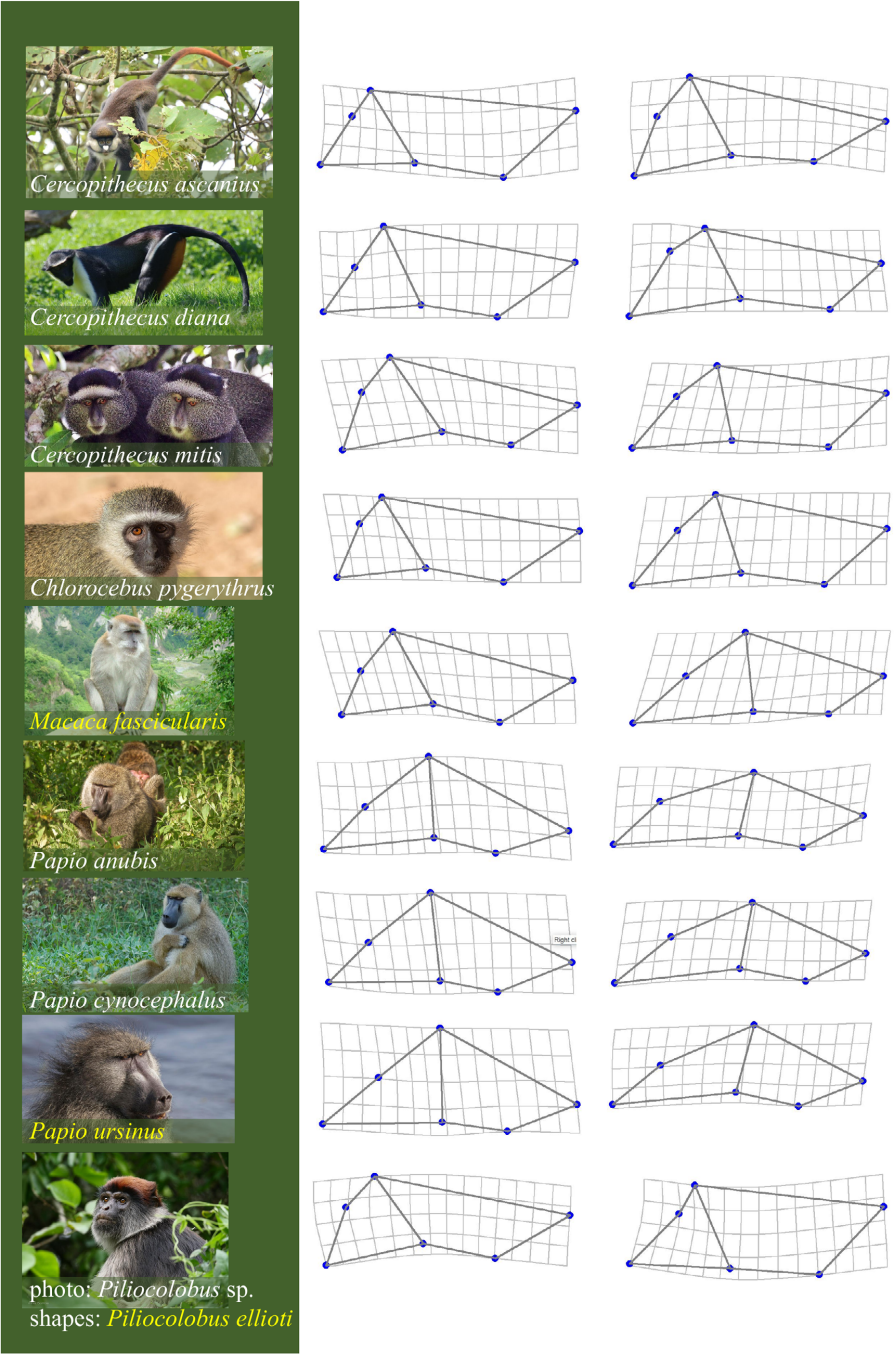
Example of non‐negligible allometric variation predicted by the multivariate regressions for the opposite extremes (magnified three times) of size variation in the largest male samples (*N* ≥ 30). Photos for the species in this analysis (with images cropped, and sex unknown except for male macaques and baboons) are from Wikimedia Commons (see below) under the Creative Commons Attribution‐Share Alike 2.0, 3.0, 4.0, except the red colobus at the bottom (by Nick Borrow, CC BY‐NC 2.0), whose species is unknown and was used as an example for this genus due to the nonavailability of a photo of *P. ellioti*. The original webpages of the photos, from the top to the bottom, are: https://en.wikipedia.org/wiki/File:Red‐Tailed_Monkey,_Uganda_(15587657375).jpg. https://upload.wikimedia.org/wikipedia/commons/5/5f/La_Bourbansais_04.jpg. https://en.wikipedia.org/wiki/Blue_monkey#/media/File:Blue_monkey_(Cercopithecus_mitis_stuhlmanni)_pair.jpg. https://upload.wikimedia.org/wikipedia/commons/4/40/Cercopiteco_verde_%28Chlorocebus_pygerythrus%29%2C_parque_nacional_Kruger%2C_Sud%C3%A1frica%2C_2018‐07‐25%2C_DD_57.jpg. https://upload.wikimedia.org/wikipedia/commons/5/53/Ngarai_Sianok_sumatran_monkey.jpg. https://en.wikipedia.org/wiki/Olive_baboon#/media/File:Papio_anubis_in_Kenya.jpg. https://upload.wikimedia.org/wikipedia/commons/2/2c/Chacma_baboon_%28Papio_ursinus_griseipes%29_male_head.jpg. https://www.flickr.com/photos/nikborrow/42455290670.

Non‐negligible allometric shape changes are exemplified for males in Figure 4 using the species with larger samples (*N* ≥ 30, as in Figure 3b). Static allometric variation is relatively modest and needs to be magnified three times to attempt a description of the main patterns. I focus on the three species of Figure 4, *M. fascicularis*, *P. ursinus*, and *P. ellioti*, where the effect of static allometry is stronger (Rsq > 10%). In the crab‐eating macaques and Chacma baboon, smaller individuals tend to be relatively less prognathic and have longer cranial vaults compared to their larger conspecifics. In the Elliot's red colobus, which has a fairly flat face, as in most colobines (Ledevin & Koyabu, 2019), the cranial vault of the smaller individuals is proportionally slightly longer. This aspect of allometric variation is similar to the pattern described in *M. fascicularis* and *P. ursinus*. In contrast, a reduced prognathism in smaller individuals of the Elliot's red colobus is less evident. In fact, the deformation grids and wireframes suggest that, in this species, a more pronounced flexion of the palate relative to the comparatively shorter cranial base might make the face of smaller individuals look less prognathic without any substantial shortening of the palate.

All analyses (*N* ≥ 30, 20, 10) in both sexes show a wide variation in interspecific angles between static allometries (Table 2). Although on average pairwise angles are approximately 50–70°, angles can vary between about 30° and 100° or more, even when the range is trimmed (q10‐q90 in Table 2) to exclude the 10% of the most extreme values at both ends of variation. Averages of the angles, as well as their ranges, are evidently larger when the species with smallest minimum *N* (*N* ≥ 10) are also included in the analysis. In contrast, within each sex, summary statistics for pairwise angles are fairly similar using either a minimum *N* of 20 or one of 30. The standard deviation of the pairwise angles (~21° on average), however, is fairly similar in all datasets regardless of the minimum N used to select the species.

### 3.2 Congruence between allometric and evolutionary divergence

**FIGURE 5:**
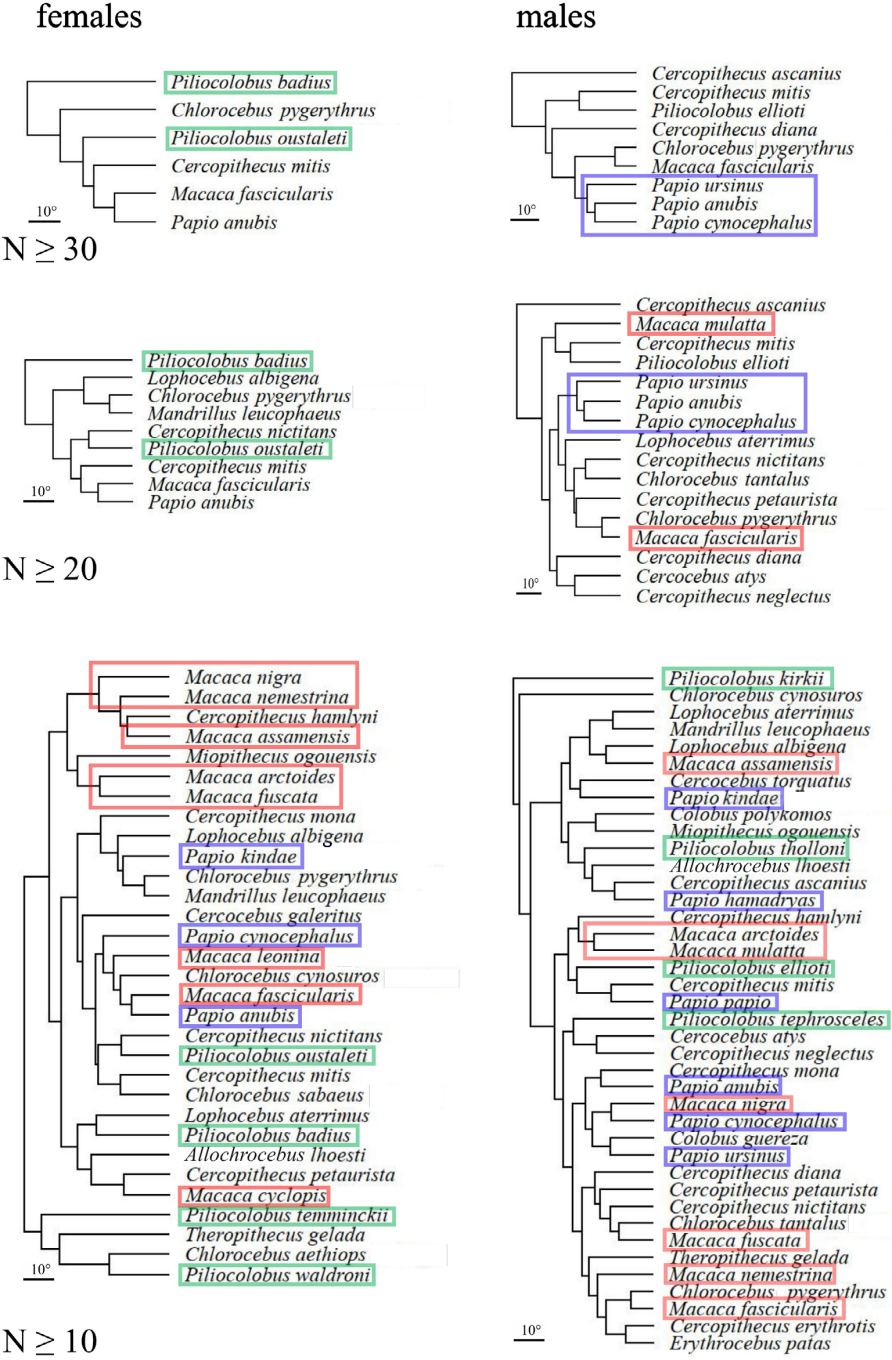
UPGMA phenograms based on angles (in degrees) between species allometric trajectories. Macaques, baboons, and red colobus are emphasized with, respectively, red, blue, and green frames to exemplify the general lack of congruence between phenetic clusters and well supported, diverse, monophyletic genera.

Given that angles between pairs of non‐negligible static allometric vectors suggest an ample range of directions, the next question is whether allometric divergence happens proportionally to evolutionary divergence. In the phenograms using angles (Figure 5), this does not seem to be the case, as the trees do not show a strong pattern of clustering according to taxonomy. This finding is consistent with the observation that, on average, angles between species of the same genus are not much smaller than between species of different genera (Table 3). In fact, the latter are no more than a few degrees larger (values emphasized with gray background in Table 3), if larger at all, and in one case (females with *N* ≥ 30) it is within‐genus angles that are wider on average than between genera. There is also no clear clustering according to tribe or subfamily, as Cercopithecini, Papionini, and Colobini are mostly mixed up in the UPGMA trees. The main exception to the almost complete incongruence between taxonomic separation and allometric divergence is male baboons in the two datasets with larger *N* (≥20 and ≥30), where the three species with non‐negligible regressions cluster together.

**TABLE 3:** Median of species pairwise angles of static allometries within and between genera (gray background emphasizes when angles are on average larger between genera rather than within).

	Median		Angles	
	Females		Males	
Min. *N*	Within^a^	Between	Within	Between
30	69	52	57	60
20	54	54	56	59
10	67	71	60	68

^a^ For females with min. *N* = 30 or 20, within genus angles are only one and two cases, respectively.

Matrix correlations between allometric angles and patristic distances calculated from the 10Ktrees chronogram generally indicate a very modest congruence between them (Table 4). For the angles, the values of *K* were also modest (<1), as they ranged, when significant, between 0.02 and 0.4 (Table 4). In two cases (females with *N* ≥30 or 20), however, both correlations and *K* were fairly high (*r* = 0.6–0.8; *K* = 0.9–1.1). Yet, analyses in these datasets, which included fewer species than in all other cases, never reached statistical significance for *K*. Thus, overall, the results suggest that there might be a phylogenetic signal in angular divergence of allometric trajectories, but the signal is tiny and never reaches significance. Interestingly, however, *K* tends to be larger when the smaller species samples are excluded, as it increases in both sexes in relation to the minimum N threshold.

**TABLE 4:** Phylogenetic signal explored using matrix correlations (pairwise angles or mean shape distances vs. patristic distances from the 10Ktrees molecular chronogram) and tested using the multivariate *K* statistics (tests with *p* < 0.005 are emphasized in italics).

			Angles			Mean Shapes		
Sex	Species *n* ^a^	Min. *N*	Matrix *r*	*K*	*p* ^b^	Matrix *r*	*K*	*p* ^b^
F	5	30	0.763	1.062	0.196	0.145	1.007	0.238
	8	20	0.575	0.873	0.053	0.273	0.924	0.132
	25	10	0.218	0.388	0.051	0.358	0.994	*0.001*
M	9	30	0.068	0.578	0.244	0.757	2.349	0.006
	15	20	0.022	0.485	0.086	0.361	1.672	*0.001*
	36	10	0.179	0.184	0.266	0.233	0.815	*0.001*

^a^ When smaller than in Table 2, it is because a few species are missing in the 10Ktrees chronogram.

^b^ *p*‐Value for the *K* statistics.

For a comparison (see Section 4), Table 4 also shows results (matrix correlations and K) using, instead of pairwise static allometric angles, the Procrustes shape distances between the mean shapes of the same species: in this case, unlike with angles and with few exceptions (the two female datasets with only 5–8 species), species mean shapes showed larger correlations and often significant *K*s.

### 3.3 Randomized subsampling experiments to explore the impact of sample size on estimates of angles between allometric trajectories

To introduce this subsection of the Results, I first go briefly back to the findings in the main set of regressions (Section 3.1). In the main analysis, the summary statistics of Table 2 suggest a trend toward larger averages (and ranges) of pairwise allometric angles when smaller species samples (minimum *N* ≥ 10) are included to increase taxonomic sampling. This seems to indicate an effect of sampling error, with inaccurate estimates of slopes in small samples generally leading to inflated angles. However, the interpretation is uncertain, because the angles SD varies little and results (Table 2) with intermediate minimum *N* (*N* ≥ 20) are similar to those with larger samples (*N* ≥ 30). Besides, since with a minimum *N* ≥ 10 the number of species analyzed is more than two‐to‐three times larger, one cannot exclude that the wider divergence in the angles is a genuine consequence of a better taxonomic sampling. The randomized subsampling experiments helped to clarify these doubts and, thus, allow a more accurate interpretation of the main results.

Estimates of angles in balanced bootstrapped random subsamples were on average 6–18° larger (Table 5) than the corresponding estimates using the total samples. The smaller the *N* in the balanced randomized bootstrapped subsamples, the larger the positive bias and the larger the range of variation in angles (larger SD, trimmed and absolute ranges for the angles). Overall, therefore, the randomized subsampling experiments strongly indicated that the increase in the observed divergence of allometries when minimum *N* ≥ 10 (Table 2) is mainly driven by sampling error and the much larger uncertainties in the smallest (10 ≤ *N* ≤ 20) species samples.

**TABLE 5:** Summary statistics for the differences in averaged angles estimated using perfectly balanced bootstrapped subsamples (100 bootstraps) and the observed angles in the same species using all individuals: Positive or negative values mean that angles are, respectively, overestimated or underestimated (the latter emphasized with a gray background) on average in the bootstrapped subsamples^a^.

Sex	Species *n*	*N*	Mean	SD	Median	q10	q90	Min	Max
F	6	30	9	5	8	3	16	1	17
	6	20	12	8	12	2	22	1	26
	6	10	18	13	16	0	33	−5	38
M	9	30	7	7	6	−1	16	−11	23
	9	20	9	10	9	0	19	−19	32
	9	10	15	15	15	0	33	−27	47

^a^ As explained in detail in Section 2, species with *N* > 30 and non‐negligible regressions (first block of Tables S1 and S2 with six female species and nine male species) are selected to build bootstrapped subsamples of *N* = 30, 20, or 10. Each bootstrapped subsample is used to replicate the allometric regression and then recompute the species pairwise angles. The median of the angles of each dataset (e.g., 100 bootstrapped female samples with *N* = 30 in all six species) is calculated and its difference from the observed angles in the total (all individuals) samples is computed pairwise. Differences (e.g., bootstrapped medians minus observed for the 15 interspecific angles in the 6 female species, etc.) are summarized using means, SD, medians, q10–q90 percentiles and the minimum and maximum (see Table 2 for the abbreviations).

## 4 DISCUSSION

I open this section by recapitulating the main results and by discussing some of the methodological issues. Later, I go on with the interpretation of the results, the main conclusions and future directions.

### 4.1 Static allometry: Effect size, significance, angles, and sampling error

How strong is static allometry in the cranium of Old World monkeys? The effect size of allometry is typically moderate (median Rsq = 10–11%). To put it into context, even if they used a larger landmark configuration, Cardini and Elton (2017) found that sexual dimorphism in guenon skulls had a median Rsq of 19%, which is almost twice that of static allometry in this study. Because the effect of static allometry is generally modest in both female and male Old World monkeys, it is not surprising that regressions often fail to reach statistical significance and the proportion of significant regressions drops when species with smaller samples are included. Larger samples not only increase power, but are more representative of population variability and likely provide a more accurate picture of size and, thus, size‐related shape variation within each species. For instance, the range of lnCS variation in females with *N* > 10 has a 0.6–0.7 Pearson correlation with species *N* (respectively, excluding or including the very large *M. fascicularis* sample). A similar correlation is found also in males (*r* = 0.7 both with or without *M. fascicularis*). These correlations indicate that the amount of variation in the samples is strongly and positively influenced by sample size and, therefore, only the largest samples are likely to approximate the true extent of cranial size and allometric shape variation in the adults of a species.

The considerations on effect size and *N* should be borne in mind to cautiously interpret also the visualization of static allometric shape variation. Figure 4 shows in most species, with differences in degree and strength, a trend toward relative facial elongation and braincase reduction, which is, as mentioned in the Introduction, a pervasive pattern of both ontogenetic and evolutionary allometry in primates and more generally in mammals (Cardini, 2019). Yet, the pattern is far from obvious in some cases, such as Elliot's red colobus and the red‐tailed guenon (*C. ascanius*), and, depending on the species, may be more evident in dorsal rather than ventral measurements of the cranium, or vice versa. Whether the aspects specific to each species are genuine or to a larger or smaller extent affected by sampling error it is difficult to say, but there is little doubt that sampling error introduces inaccuracies. I further discuss this issue in the next paragraphs on Rsq and angles, but those considerations apply also to the visualization of allometries, because allometric shape diagrams are also computed using the regression coefficients.

In relation to the choice of the minimum *N* for the inclusion of species samples in the analysis, the multivariate regressions showed reduced power (a smaller proportion of significant analyses) and a small inflation of Rsq (becoming on average larger) when the N threshold was lowest and smaller samples were included. With more species and, thus, a better taxonomic sampling, the range of results (both Rsq and angles) also widened remarkably (Table 2 and Tables S1 and S2). Regrettably, this wider range is, as anticipated at the end of the Results, likely mostly a consequence of sampling error rather than being an accurate outcome of the better taxonomic representativeness of the analyses that included smaller samples and, thus, more species. The inflation of Rsq (both on average and in terms of variability) in small samples is both theoretically expected (Cramer, 1987; Wainer, 2007) and supported by previous studies using multivariate regressions (Cardini & Elton, 2007). Randomized subsampling experiments using a large configuration of skull landmarks in vervets (Cardini & Elton, 2007) showed that, when the most accurate estimates of Rsq for static allometries was ~17–19% ± 3% (total *N* ≥ 100), small samples (*N* = 10) produced mean estimates of 22–32% ± 6% or more. Thus, as in this study, Rsq was inflated and the range of its estimates became wider as *N* was reduced. These observations further stress the importance of large samples in studies at the boundary between micro‐ and macro‐evolution (Cardini et al., 2021). Indeed, in the balanced randomized bootstrapped subsamples, even when *N* = 30, divergence in static allometries was on average overestimated compared to the angles calculated using the total samples. This indicates that not even 30 adult females (or males) per species may be enough for accuracy.

The balanced randomized bootstrap subsampling experiments not only showed that angles are overestimated on average (+6–16°) in smaller samples, but also confirmed that the range of estimates is wider. For instance, the SD of the differences between angles from bootstrapped subsamples and observed total samples more than doubled from *N* = 30 to *N* = 10 (Table 5). Because in smaller samples pairwise estimates of angles vary more, it is worth observing that, despite an average overestimate when *N* is reduced, some of the angles (negative deviations emphasized with a gray background in Table 5) can be smaller than observed in the total samples. This is a useful warning not to expect that smaller samples will always inflate angles between static allometric vectors: on average they will, but in some cases one might in fact get an underestimate. As with the Rsq, the observations on the impact of N on estimates of static allometric divergence are, again, congruent with previous work on crania, which suggested the need of really large samples for accuracy and precision of static allometric regressions in primates (and likely most mammals). Specifically, Cardini and Elton (2007) found that approximately 60 individuals per species were necessary to have estimates of allometric angles on average within 50% of the most accurate result obtained in their total samples of vervets and blue monkeys.

As in Cardini and Elton (2007), the randomized subsampling experiments in this study were run using perfectly balanced samples. This was done to be confident that the effect on estimates of angles was exclusively driven by sample size. As it is generally the case with other statistical analyses, variability in sample size across groups typically contributes to inaccuracies, reduces robustness and makes interpretations less simple. Yet, as shown in my own dataset, heterogeneous *N*, with often many relatively small samples, is every‐day life for those working on wild species and especially when they must rely on museum collections. These are a most precious source of information, but also one where researchers opportunistically depend on what is available, as well as in relation to the funds they have for visiting multiple collections. Thus, the impact of sampling error on allometric analyses is, as in taxonomy, likely to be very strong, a problem that should be acknowledged and explored (Cardini, 2020; Cardini et al., 2021). With ontogenetic data, given the much larger changes happening during growth and development, the requirement of very large samples might be less strict, but issues with power may nevertheless occur (Brown & Vavrek, 2015) and data should be equally representative of all age classes. With primates and other mammals, however, it is often the case that ontogenetic series are incomplete and the younger age groups poorly represented.

To conclude the discussion on the impact of sample size, I briefly touch on a related issue, which is not part of this study and, to my knowledge, has been rarely investigated in the context of allometry. A large p / N ratio, where p is the dimensionality of the data, is well known to be problematic in multivariate data analysis (Hair et al., 1998), so much so that even exploratory methods for dimensionality reduction might be impacted (Björklund, 2019; Cardini et al., 2019; Rohlf, 2021). Data dimensionality also has an effect on estimates of angles between vectors so that, with larger p, estimates of angles tend to cluster more tightly around the true estimate (Watanabe, 2022). Thus, in highly dimensional spaces, one can find that large angles, despite suggesting different directions of allometric trajectories, can be significantly smaller than expected for uncorrelated, orthogonal, random vectors. If a test is accurate and has a correct type I error rate (the rate of false positives), the result is valid, with more similarity in direction than predicted for random vectors. Yet, this might mean that a researcher could be pushed to interpret almost orthogonal vectors (e.g., 86° in the empirical example used by Watanabe, 2022) and, thus, seek uncertain similarities in the visualization of shape change along the corresponding trajectories. In this respect, a small landmark configuration focusing on specific but relevant dimensions in relation to a well‐defined hypothesis may help to reduce noise (aspects uninteresting in the context of that hypothesis), have a smaller dimensionality (without recurring to, for instance, using a subset of the first PCs to control for p) and lead to simpler, but potentially more robust interpretations of allometric change.

### 4.2 Allometric directions and phylogenetic divergence

Studies on static and ontogenetic allometries in terrestrial vertebrates using geometric morphometrics are numerous (e.g., Chiozzi et al., 2014; Djurakic & Milankov, 2020; Drake & Klingenberg, 2008; Le Verger et al., 2020, 2024; Marcy et al., 2020; Murta‐Fonseca et al., 2019; Simons & Frost, 2021; Singleton, 2002), but few specifically focused on the relationship between phylogeny and allometric divergence. I mainly discuss geometric morphometric analyses because methods and results are more directly comparable. My own interest was prompted by a recent publication using landmark‐based Procrustes methods in armadillos. In that work, the authors found congruence between cranial allometric divergence and taxonomic groups, so that “the greater the phylogenetic distance between species, the greater the difference in their ontogenetic and static trajectories of allometric variation” (p. 14, Le Verger et al., 2024). As mentioned in the Introduction, it might seem intuitive that, as speciation happens and evolutionary divergence increases, allometric patterns also progressively diverge in direction. Yet, phenotypic and phylogenetic differences are often not so tightly coupled and, in Old World primates, congruence with allometric divergence might be the exception rather than the rule. Simons et al. (2018) found that in cercopithecine monkeys cranial ontogenetic trajectories carry no phylogenetic signal. Singleton (2002), using cranial landmarks to compare static allometries of adult papionins, reported (p. 547) “positive facial allometry and negative neurocranial allometry,” but found a mix of divergence and homogeneity in allometric trajectories. Her results, however, are not easy to compare with mine, because she did not perform multivariate regressions and did most of her analyses with pooled sexes.

The approach I used in this study has, as anticipated in Section 2, similarities to Simons et al. (2018) and especially to Watanabe and Slice (2014). Watanabe and Slice employed a battery of analyses including matrix correlations, phenograms and tests of the phylogenetic signal (such as the univariate *k*) to investigate the phylogenetic signal in the ontogenetic allometries of crocodylians. Again, results were very similar to mine, since they also found no appreciable phylogenetic signal. Likewise, previous research on a smaller group of crocodylians suggested little congruence of ontogenetic allometric divergence with phylogeny, but, in that paper, the accuracy of the molecular phylogeny itself was subject to debate (Piras et al., 2010). This is an important point to consider, as some of the incongruence I and others have found between allometric and evolutionary divergence may in fact be due to uncertainties in the phylogenetic and taxonomic relationships. Yet, in my study, at least the monophyly of species‐rich genera such as *Macaca*, *Papio*, and *Piliocolobus* is robust. Therefore, if allometric divergence increased with phylogenetic distance, one would at least expect clustering according to taxonomy in these genera. This prediction is clearly refuted by an almost consistent lack of unequivocal clusters of congeneric species in the UPGMA trees of static allometric angles (Figure 5). Thus, it seems that, if there were similarities between closely related species when they started diverging from a common ancestor, these were mostly lost as differences increased over a longer evolutionary time span.

**FIGURE 6:**
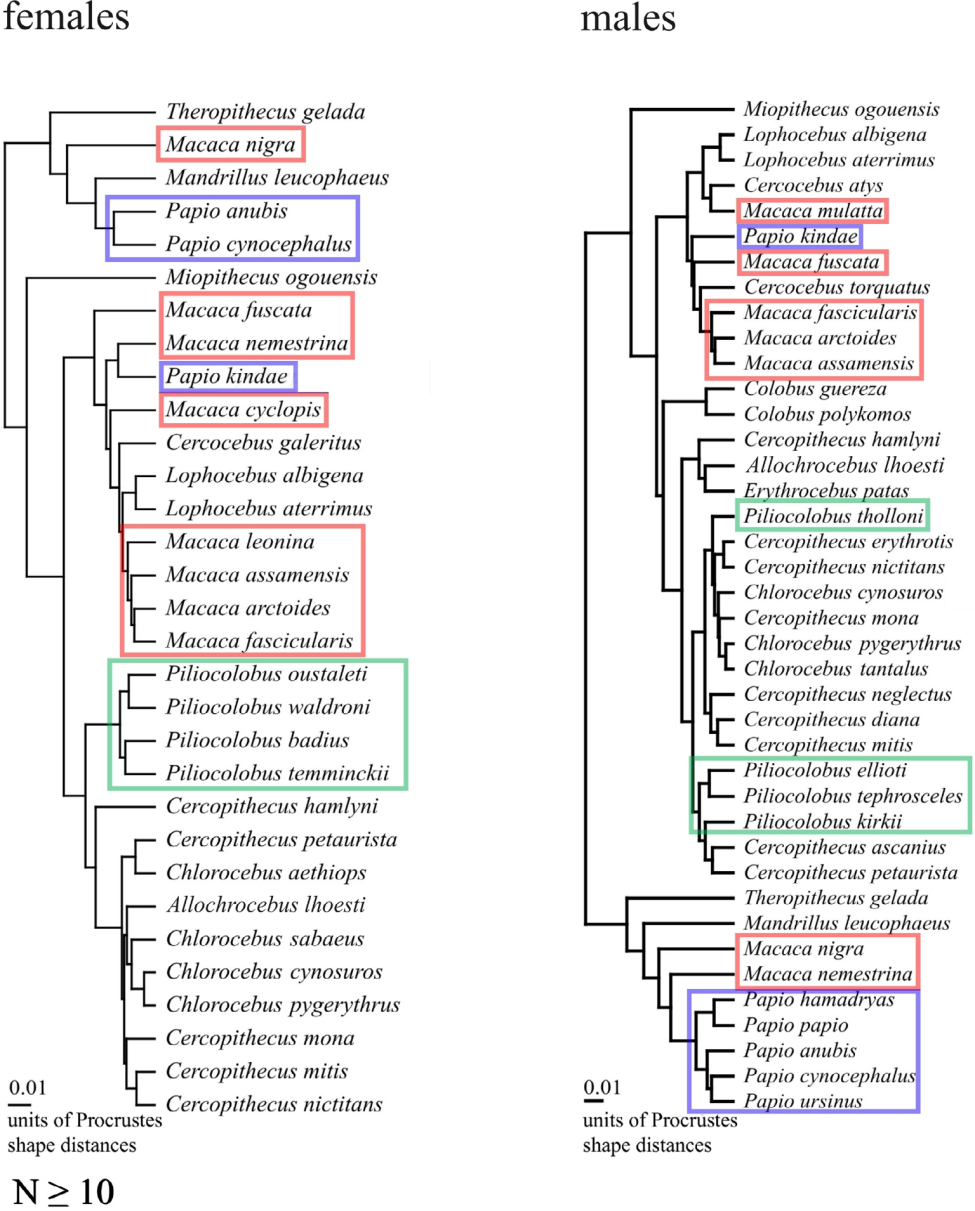
UPGMA phenograms based on Procrustes shape distances between species mean shapes (minimum *N* ≥ 10); example genera are emphasized as in Figure 5.

To explain the general lack of congruence between allometric directions and phylogenetic distances, Watanabe and Slice (2014) offered several non‐mutually exclusive explanations. Their explanations, complemented with the discussion on uncertainties in the phylogeny (above) and sampling error (Section 4.1) are valid for my analyses as well. To start, as Watanabe and Slice (2014) pointed out, we cannot exclude that the statistical model used for the analysis is inadequate. This is, of course, true for all statistical analyses. For instance, in morphometrics we usually prefer linear models for their simplicity, but allometries could be nonlinear or multiphasic (Kluge & Strauss, 1985). Together with the statistical model, we should also consider that what is being measured is crucial, as I have already stressed. In my study, one could argue that the problem was, in fact, one of measuring fewer landmarks than needed. I have already clarified the reasons for the choice of a simple midplane configuration. However, that even those few landmarks can be phylogenetically informative is shown by the analysis of the phylogenetic signal in species mean shapes (Table 4). Unlike most of the results for allometric angles, mean shape data showed a degree of correlation with phyletic distances and often had significant *K*s. A modest but appreciable phylogenetic signal in mean shapes is also suggested by the UPGMA phenograms of Figure 6, which include the same species as in the two trees at the bottom of Figure 5 (i.e., species with non‐negligible allometries and *N* ≥ 10). Unlike the cluster analyses of allometric angles (Figure 5), the summary of similarity relationships between species mean shapes in Figure 6 is characterized by at least a degree of taxonomic clustering, that happens despite likely large inaccuracies in estimates of species means in the smaller samples (Cardini et al., 2021).

Why, then, in contrast to species mean shapes, does the divergence of Old World primates allometries have almost nothing to do with phyletic relationships? Watanabe and Slice argued that, in crocodylians, allometric patterns tend to be so evolutionarily labile that the phylogenetic signal is soon lost, a point made also by Simons et al. (2018) and Pavón‐Vázquez et al. (2022). This explanation, if valid for my study on Old World monkeys, raises an interesting question: if allometry really varies so easily in direction, is there a contradiction with the observation of the conserved “CREA” pattern of evolutionary allometric change in cercopithecids (Cardini & Polly, 2013; Radinsky, 1985)? I do not think there is a contradiction, when the problem is carefully considered. First, the level of the analyses (within species vs. above species) is different. More importantly, a macroevolutionary trend, such as the positive allometry of the snout and the negative allometry of the braincase, may be found in closely related species and also have broad similarities to within species patterns. Yet, there may be differences in many, less evident, aspects. It is these differences in “details,” caused by a combination of stochastic and selective forces (plus, possibly, plasticity), which likely amplify allometric divergence during evolutionary radiations in which, as in the Old World monkeys, a variety of geographic regions, habitats and ecological niches are colonized (Elton, 2007; Frost, 2017; Lo Bianco et al., 2017). Indeed, although the common mammalian pattern of relative facial elongation and braincase reduction in larger species was well supported in the same lineages I have analyzed in this article, with slopes of regressions of facial length onto braincase length mostly larger than one (Cardini, 2019), evolutionary allometric slopes varied widely among clades. Part of the variation was, as in the present study, a likely consequence of statistical uncertainty (sampling error, different tree topologies in comparative analyses, etc.), but another part likely reflected genuine taxonomic differences. Thus, the general shape change predicted by CREA can occur in a lineage, but the precise details vary from case to case.

### 4.3 Static versus ontogenetic trajectories

Static allometry in adults captures only a specific aspect of the very end of a much longer and complex ontogenetic path. If developmental changes contribute to species divergence, a fuller quantitative description of ontogeny (allometry, development, growth) is crucial. Ideally, one might want to use longitudinal data, rather than cross‐sectional, and both pre‐ and post‐natal information. Unfortunately, as already discussed, in most primate species and, more generally, in mammals with few exceptions, the availability of representative samples covering all main age classes is a rare instance even when one is happy to use post‐natal cross‐sectional data. Simons and Frost's (2021) analysis of cranial ontogeny in catarrhines, for instance, includes relatively few representatives of this diverse group and, as they acknowledge, samples may be inadequate in several cases. For this reason, as in previous work, they (Simons et al., 2018; Simons & Frost, 2021) pooled sexes, arguing that this is a reasonable approximation, because within species ontogenetic trajectories diverge between sexes only toward the end of development. But this is also a compromise, as the degree of sexual dimorphism varies from species to species (Gordon, 2006; Smith & Cheverud, 2002) and that implies a different amount of divergence in older ontogenetic stages, which may be negligible in some species and not in others. This might be a small source of error and most of Simons and colleagues' findings, at least those concerning the divergence of ontogenetic trajectories, are probably robust in their conclusions. Yet, it is another example of how difficult it is to perform accurate allometric studies in primates.

In spite of the limitations I have briefly discussed, Simons and Frost (2021) is to date possibly the largest geometric morphometric study of cranial ontogeny in catarrhines. As three of the four lineages they analyzed (i.e., papionins, cercopithecins, colobines, and apes) are the same as in my study, their work is particularly important to appreciate the potential differences between ontogenetic and static patterns of size‐related shape variation in Old World monkeys. A long quote from the main conclusions of Simons and Frost (2021) (p. 703) is useful to compare our results: “In general … the amount of shape change (i.e., the length of the trajectory) over ontogeny from the time of the full eruption of dP4 to adulthood is mostly conserved among catarrhines, whereas differences in the pattern of shape change (i.e., the direction measured using pairwise angles) over ontogeny are common … However, within each of the four subclades, allometric trajectories were largely oriented in the same direction … This indicates that one of the ways catarrhines are achieving differences in adult cranial morphology is through relatively slight, though detectable differences in the patterns of those changes, but not through truncation or extension of a common pattern. These modest differences in patterns of ontogenetic shape changes are therefore adding to the well documented pre‐natal and early post‐natal differences.” Therefore, while Simons and Frost (2021) found divergence in patterns of cranial ontogeny, they also stated that the species‐specific trajectories largely point in similar directions within the four catarrhine clades. This seems in stark contrast compared to my results of remarkably divergent static allometries. However, for a more accurate comparison, I computed the median allometric angle in the colobines, cercopithecins, and papionins using the table published by Simons and Frost (2021). Within each of the three lineages, the median angle is approximately 20°−25° (with an average trimmed range—q10 to q90—of 15° to 30°). These angles are approximately half or less than found in my analysis of static allometries in same taxa, where median angles were larger than 50° and ranged (q10–q90) from about 30° to more than 100°. Thus, the difference is indeed large and even more impressive if we consider that, when the same species was analyzed, my estimate of an allometric trajectory corresponds to the end part of the regression line computed by Simons and Frost. Then, what is the reason for such a clear dissimilarity between the average divergence of ontogenetic and static allometries?

The answer is not easy, but some speculations are possible. Taxonomic samples are not identical, even if most of the species of Old World monkeys analyzed by Simons and Frost are also present in my study. Sample composition, however, cannot explain on its own the large incongruence we observe, since even the upper limit of their range of angles (q90–30°) barely overlaps with the lower limit of my estimates (q10–30°). In contrast, as mentioned, pooling sex likely introduced some inaccuracy in the estimates of ontogenetic divergence of Simons and Frost (2021). It is hard to say with confidence whether that led to a degree of underestimation of angles of ontogenetic trajectories. In contrast, I have almost certainly inflated estimates of divergence, because of the stronger effect of sampling error on the much shorter static allometries. Thus, to some extent, the different results in the study by Simons and Frost (2021) and this study probably relate in part to issues with samples. It is also likely that ontogenetic analyses capture larger changes whose direction might be less affected by “details,” which in contrast matter relatively more when less variance is accounted for by a factor, as it happens with static allometry. Thus, species‐specific aspects of size‐related shape change could be a major contributor to divergence in static allometries, whereas small changes that deviate from a main common trend in large ontogenetic variation could have a more modest impact on allometry (at least when modeled with a simple linear regression). Likewise, as discussed in the previous subsection, the much larger interspecific allometric variation across closely related species of Old World monkeys is one of the probable reasons why CREA is supported in these lineages (Cardini, 2020) despite a huge variability in the direction of their static allometries. Another, more obvious, reason for differences in results between my study and the ontogenetic analyses of Simons and Frost (2021) is that the landmark configuration is not the same, with Simons and Frost measuring at least four times (considering only one of each pair of their bilateral landmarks) the number of landmarks of my study. With fewer landmarks, the range of estimates is likely to be wider, but the average should not be biased. Regardless of the explanation, on a practical level, finding large differences between estimates of allometric divergence in static and ontogenetic analyses does not induce optimism in the possibility of accurately using static allometries as a proxy for ontogenetic ones, when subadults and young are not available in a study. This is another aspect that deserves careful scrutiny in future research on allometry.

Despite the evident difference in the amount of allometric divergence in allometries, however, both Simons and colleagues and I discovered almost no phylogenetic signal in size‐ or age‐related within species shape trajectories. Their tests for *K*, like mine, were non‐significant. Furthermore, if one computes median angles within and between genera using the matrix of interspecific pairwise angles of ontogenetic change of Simons et al. (2018), the angles between species of different genera are on average only slightly larger than those between species of the same genus (27° vs. 24°, respectively). This similarity in the average divergence within and between genera is in good agreement with my results (Table 3) and, together with the nonsignificance of *K*, corroborates the conclusion that there is hardly any congruence in Old World monkeys between phylogenetic structure and allometric divergence regardless of using static or ontogenetic data. Thus, the hope that differences in direction of within‐species trends in cranial shape change may help to elucidate the phylogeny of living and extinct species, in primates and other mammals, as well as in the context of human evolution, seems to be small. With fossils, the problem is further aggravated by the incomplete and generally scarce study material of most species.

### 4.4 Conclusions and future directions

Static allometries encompass relatively little variation and have a small effect size. Estimates may require samples close to or larger than 100 individuals per species and sex (see fig. 3a,b of Cardini & Elton, 2007), before a level of accuracy appropriate for assessing a potential phylogenetic signal is achieved. Ontogenetic trajectories may need smaller samples, but only as long as variation in all age classes is truly representative. Also, as briefly discussed, and especially in ontogeny, one cannot exclude that change is multiphasic, so that, for instance, the slope of a regression varies depending on the stage. For ontogenetic variation, we should more often consider nonlinearities. Trajectories can be split into subsets of comparable ontogenetic stages (Neubauer et al., 2010; Scott et al., 2014). Alternatively one can explore the application of a multivariate curvilinear regression using a polynomial expansion of the size predictor. A similar approach has been shown to be useful for modeling clinal variation in primates (Cardini et al., 2010). When a more sophisticated method, with more parameters, is employed, however, sample representativeness is even more crucial, and over‐fitting must be excluded before the model is selected. As in biogeography (Cardini et al., 2010), resampling methods and the use of information criteria may help to estimate uncertainties and compare models.

If the change in direction of allometry in Old World primates is unlikely to be phylogenetically informative, that does not diminish the relevance of size‐related shape changes in the study of morphological variation in primates and other tetrapods. Cardini and Elton (2008b) argued that allometry is a major player in the cranial evolution of guenons and that, if size is more labile than non‐allometric shape, this contributes to the weak phylogenetic signal in the mean cranial shape differences of these species. However, this is not the same as saying that allometry is either the main or an exclusive driver of morphological change. That allometry might have acted, in guenons, as a path of least evolutionary resistance does not imply a simple extension/truncation of ontogenetic allometry. Likewise, it does not mean that allometry explains most morphological differences: “size … has helped to determine morphological change … biasing of the direction of evolutionary shape modification … [but] Such biases … decay with time, and shape variation is progressively less constrained as the structure of trait covariance is modified by selective pressures … Ecology and evolutionary history remain important components in understanding … morphological differentiation … [with] instances in which morphological divergence cannot be attributed mostly to size” (pp. 633‐634, Cardini & Elton, 2008c).

Species‐specific differences can occur together with, and in spite of, broad similarities in patterns of CREA (Cardini, 2019, and references therein). Allometry is not a unsurmountable constraint that narrowly limits morphological evolvability. Thus, for instance, CREA has exceptions such as saber‐tooth cats (Tamagnini et al., 2023) and probably our own lineage, the hominins (Cardini & Polly, 2013). Even when the evidence for CREA is robust, so that larger species tend to be more prognathic than their smaller close relatives, the trend explains only a fraction of cranial variation and does not exclude the presence of taxon‐specific aspects of allometric change (Cardini, 2019; Marugán‐Lobón et al., 2022).

What are the future directions for allometric analyses trying to bridge micro‐ and macro‐evolution in primates and other vertebrates? The issue with sampling error remains crucial, as I have stressed multiple times. Also, there is the question of what is being measured and, in relation to this, whether findings have external validity. The choice of a small set of landmarks tailored to the specific allometric hypothesis being investigated may be wiser than trying to measure everything, regardless of relevance, homology and accuracy, as in “high‐density morphometrics” (Cardini, 2020). Thus, my findings in this study are specific to a configuration of midplane landmarks mainly aimed at capturing the relative lengths of the face and braincase, an aspect central to ontogenetic development (Smith, 1997; Usui & Tokita, 2018), as well as to the study of the macroevolutionary allometry in terrestrial vertebrates (Bardua et al., 2021; Cardini & Polly, 2013; Krone et al., 2019; Marugán‐Lobón et al., 2022; Radinsky, 1985). However, there are alternatives that could be tried with a deeper understanding of primate anatomy and cranial development. Clearly, results may be different using other landmarks, focusing on different cranial regions or on another anatomical structure. Using the same configuration and species, but replicating the analysis with estimates of ontogenetic trajectories could also lead to different outcomes, but the comparison with the work of Simons and colleagues suggests that, despite their finding of a much smaller divergence, the conclusion of a negligible phylogenetic signal is probably robust. Nonetheless, if allometric change is accurately measured and detailed ecological data are available, then it might become interesting to also explore whether ecology influences the direction of allometries, as exemplified by Pavón‐Vázquez et al. (2022) using a comparative phylogenetic regression of slope coefficients onto a set of predictors measuring habitat use. Avenues for future research are vast with interesting challenges that may often concern the quality of the data more than the specific method being used. Much has been learnt, but in many respects we are still at the beginning of a deeper understanding of the relationship between shape and size and, for now, I must concur with Pélabon et al. (2014, p.71) that “the evolution of allometry … remain[s] a mystery.”

## AUTHOR CONTRIBUTIONS

**Andrea Cardini:** Conceptualization; investigation; writing – original draft; methodology; validation; visualization; data curation; formal analysis.

## Supporting information


**DATA S1:** Supporting Information.
